# Arbitrary polarization control with a segmented APPLE-II undulator

**DOI:** 10.1107/S1600577525010641

**Published:** 2026-01-01

**Authors:** Kento Inaba, Yoshiyuki Ohtsubo, Akane Agui, Hiroaki Kimura, Keisuke Sakuraba, Koji Horiba, Miho Kitamura, Shuhei Obara, Takeshi Nakatani, Tomoyuki Takeuchi, Yuji Hosaka, Masamitu Takahasi

**Affiliations:** a NanoTerasu Center National Institutes for Quantum Science and Technology (QST) Sendai Miyagi980-8572 Japan; b NAT Corporation, Hitachinaka, Ibaraki312-0005, Japan; University College London, United Kingdom

**Keywords:** APPLE-II undulator, segmented undulators, polarization control

## Abstract

A newly developed four-segmented APPLE-II undulator enables arbitrary polarization control in the soft to tender X-ray region. The undulator is installed at NanoTerasu BL13U, aiming for X-ray absorption spectroscopy over a wide energy range, 180–3000 eV, with versatile photon polarizations. By adjusting the phase difference of the synchrotron radiation using electromagnetic phase shifters, arbitrary orientations of linear polarization were obtained from left and right circularly polarized radiation. Elliptically polarized radiation was also generated from vertical and horizontal linearly polarized radiation. In addition, the ellipticity angles were successfully controlled. Circularly polarized radiation in the tender X-ray region is considered to be achieved by utilizing the third harmonic. Methods and performance of polarization control of the segmented APPLE-II undulator using the phase shifters are presented.

## Abstract 2

A four-segmented APPLE-II undulator was constructed, and its operation has started at NanoTerasu BL13U. The performance of arbitrary polarization control using phase shifters is demonstrated.

## 1. Introduction

Polarization control of synchrotron radiation (SR) is a key experimental technique for revealing the electronic and spin-orbital structures of materials (Hirohata *et al.*, 2020 ▸; van der Laan & Figueroa, 2014 ▸). Measurements of the X-ray magnetic circular/linear dichroism (XMCD/XMLD) are typical examples of utilizing the polarization of SR. Since the transmittance or reflectance of optical phase retarders is impractically low in the soft X-ray region, various insertion devices have been designed to control the polarization. In the soft X-ray region, grazing-incidence optical systems preserve the polarization state almost perfectly, allowing the polarization produced at the light source to be delivered to the sample without disruption. In that sense, this wavelength region offers a field where insertion device scientists can fully demonstrate their expertise. APPLE type undulators proposed by Sasaki and coworkers (Sasaki *et al.*, 1993 ▸; Sasaki *et al.*, 1994 ▸; Sasaki, 1994 ▸) are widely used to provide various polarizations such as linear horizontal, vertical, and left and right circular, by moving their magnet arrays mechanically along the beam axis.

Another way for polarization control is the use of magnetic phase shifters between tandem undulator segments, originally proposed by Kim (1984 ▸) as a cross undulator. The tandem undulators are arranged with orthogonal magnetic fields, and magnetic chicanes are introduced by the phase shifters to delay the electrons. The relative phase of SR from the undulators is adjusted by tuning the chicane field. The polarization state is switched through the interference between the radiation. In Kim (1984 ▸), this method was proposed to generate arbitrarily adjustable elliptical polarization from horizontal and vertical planar undulators, though it was also applied to generate linearly polarized light from left and right helical undulators (Tanaka & Kitamura, 2002 ▸).

Polarization control by the phase shifters offers two advantages over the switching method based on the mechanical movement of magnet arrays. The first advantage is that circularly polarized light of higher harmonics can be available. It is well known that helical undulators generate only the fundamental radiation when observed on axis (Kincaid, 1977 ▸); however, we can extend the available energy range for the circularly polarized radiation by interfering with the higher harmonics from linear horizontal and vertical undulators (Matsuda *et al.*, 2019 ▸). The second advantage is that faster polarization switching can be achieved. If we design a phase shifter using electromagnets, fast polarization control can be realized since the chicane field is adjusted only by changing the coil current.

One successful example of such an undulator is the segmented cross undulator (SCU) installed at SPring-8 BL07LSU (Yamamoto *et al.*, 2014 ▸; Miyawaki *et al.*, 2021 ▸). The SCU consists of a total of eight segments of tandem-arranged vertical and horizontal figure-8 undulators (Tanaka & Kitamura, 1995 ▸; Tanaka & Kitamura, 1996 ▸), which enable switching between left circularly polarized (LCP) and right circularly polarized (RCP) light as well as ±45° linearly polarized light by the phase shifters. It has also been reported that the rotation of linear polarization was achieved by superposing LCP and RCP radiation generated from two separate sets of four segments (Kudo *et al.*, 2021 ▸). However, when the number of seed segments is reduced, the achievable degree of polarization degrades (Tanaka & Kitamura, 2002 ▸). Although the obtained degree of polarization was not mentioned in Kudo *et al.* (2021 ▸), our numerical calculation using the simulation code *SPECTRA* (Tanaka, 2021 ▸) and the undulator parameters (Yamamoto *et al.*, 2014 ▸; Miyawaki *et al.*, 2021 ▸) predicts that the degree of polarization is 0.6 at most for 400 eV photons. Since the number of seed segments was essentially one for both the LCP and the RCP radiation, the achievable polarization degree was not high.

To maximize the degree of freedom in polarization control and achieve fast polarization switching, we newly constructed four-segmented APPLE-II undulators with electromagnetic phase shifters at NanoTerasu BL13U. A conceptual design of the undulator is already published in Matsuda *et al.* (2019 ▸). BL13U was designed for X-ray absorption spectroscopy over a wide energy range of 180–3000 eV (Ohtsubo *et al.*, 2022 ▸; Ohtsubo *et al.*, 2025 ▸). By adopting APPLE-II for each undulator segment, it becomes possible to generate linearly polarized light in arbitrary directions from LCP and RCP photons, as well as elliptically polarized light from the vertical linearly polarized (VLP) and horizontal linearly polarized (HLP) photons in a simple way. In this paper, we demonstrate results of arbitrary polarization control using phase shifters not only for the fundamental but also for the third harmonic.

## 2. Undulator specification

**Figure 1:**
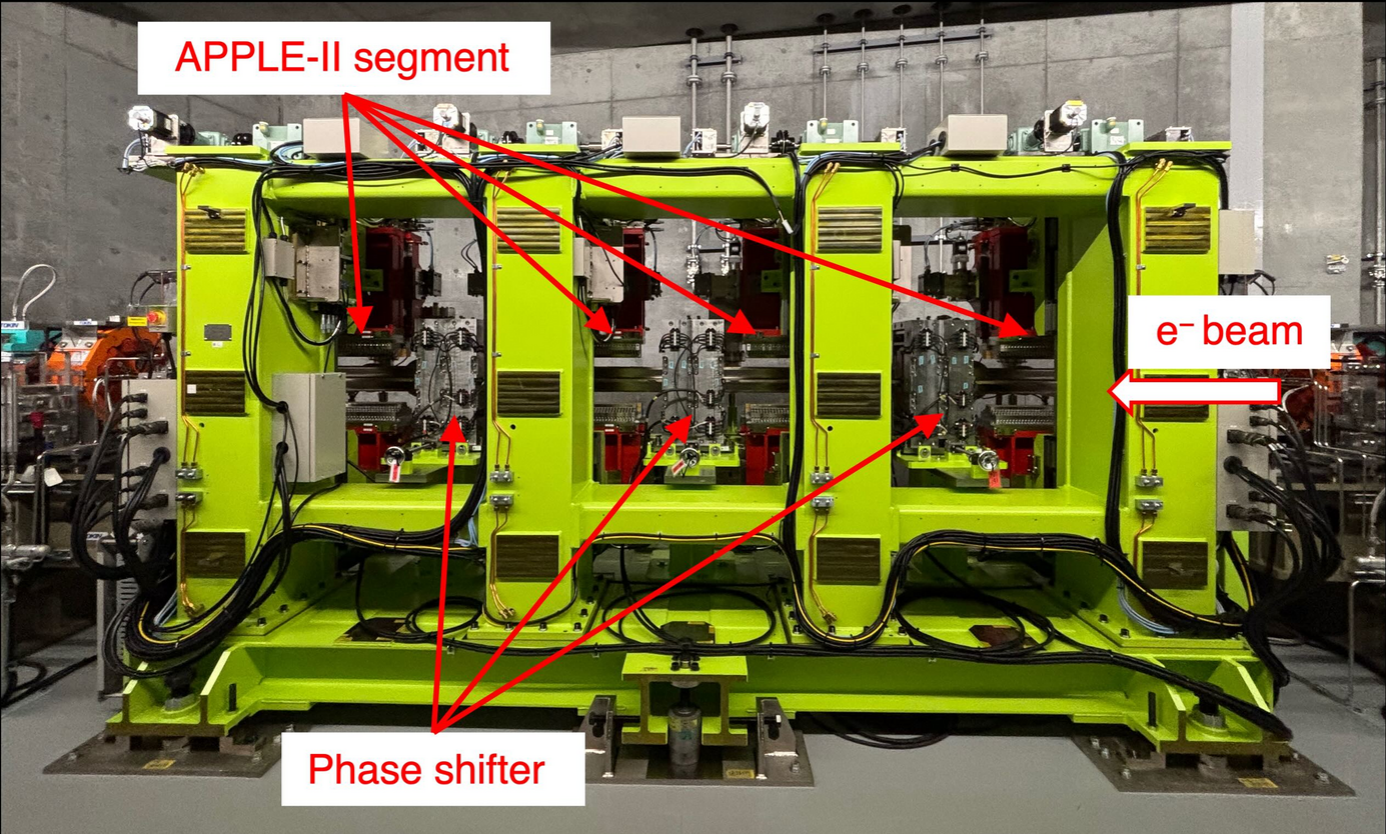
Photograph of the segmented APPLE-II undulator installed at BL13U, NanoTerasu.

Fig. 1 ▸ shows the four-segmented APPLE-II undulator at NanoTerasu. Four short APPLE-II undulator segments are tandemly arranged on a common 4 m-long frame. Each undulator segment has 10 periods with a periodic length of 56 mm. TiN-coated NdFeB alloy with remnant induction 1.3 T is used for the magnets. The maximum deflection parameters at the minimum gap of 15 mm are *K*_*x*_ = 3.3 (vertical linear) and *K*_*y*_ = 4.7 (horizontal linear). For the circular polarization, the deflection parameter at the minimum gap is *K* = 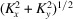 = 3.8. Table 1 ▸ summarizes the undulator parameters.

**Figure 2:**
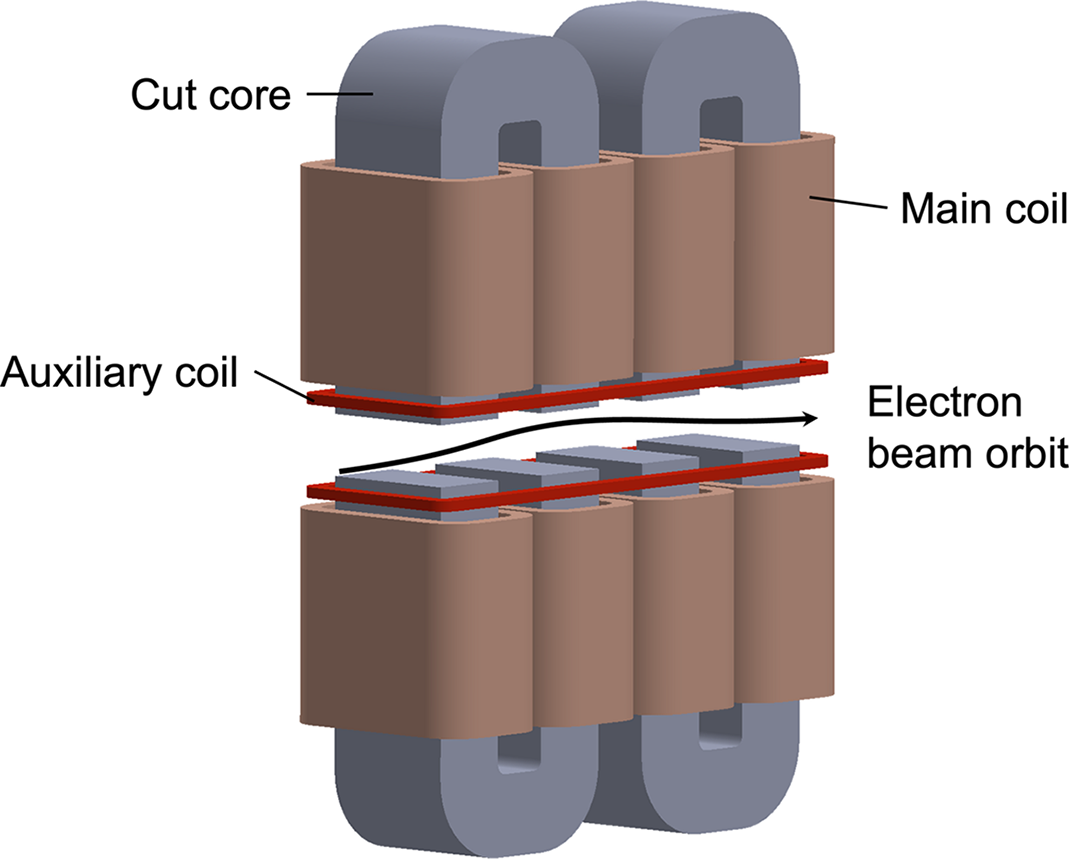
Schematic of electromagnetic phase shifter. The arrow depicts an example of the electron beam orbit passing through the phase shifter.

Three electromagnetic phase shifters are installed between each undulator segment on the common frame. Fig. 2 ▸ shows a schematic of the electromagnetic phase shifter. The phase shifter is composed of four cut-core coils. The size of the phase shifter, including its frame, is 280 mm along the beam axis, 330 mm horizontally and 570 mm vertically. The core material is a 0.025 mm laminated amorphous alloy. The main coil has 38 turns, and the four coils are connected in series. The inductance evaluated at 120 Hz with the four coils is 15 mH. A unipolar power supply with a maximum rating of 20 A is used. A symmetric magnetic field is generated on the beam axis by applying the coil current. A field strength of 0.1 T is obtained at 20 A, which corresponds to a phase shift of 11π for a 700 eV light, for example. Auxiliary coils are installed to surround the upper and lower main coils. The auxiliary coils have 3 turns and are connected to bipolar power supplies rated ±6 A. A field strength of 1 mT is generated on axis at 2 A.

The auxiliary coils are used to correct horizontal dipole kicks and to align the optical axis between the undulator segments.

**Figure 3:**
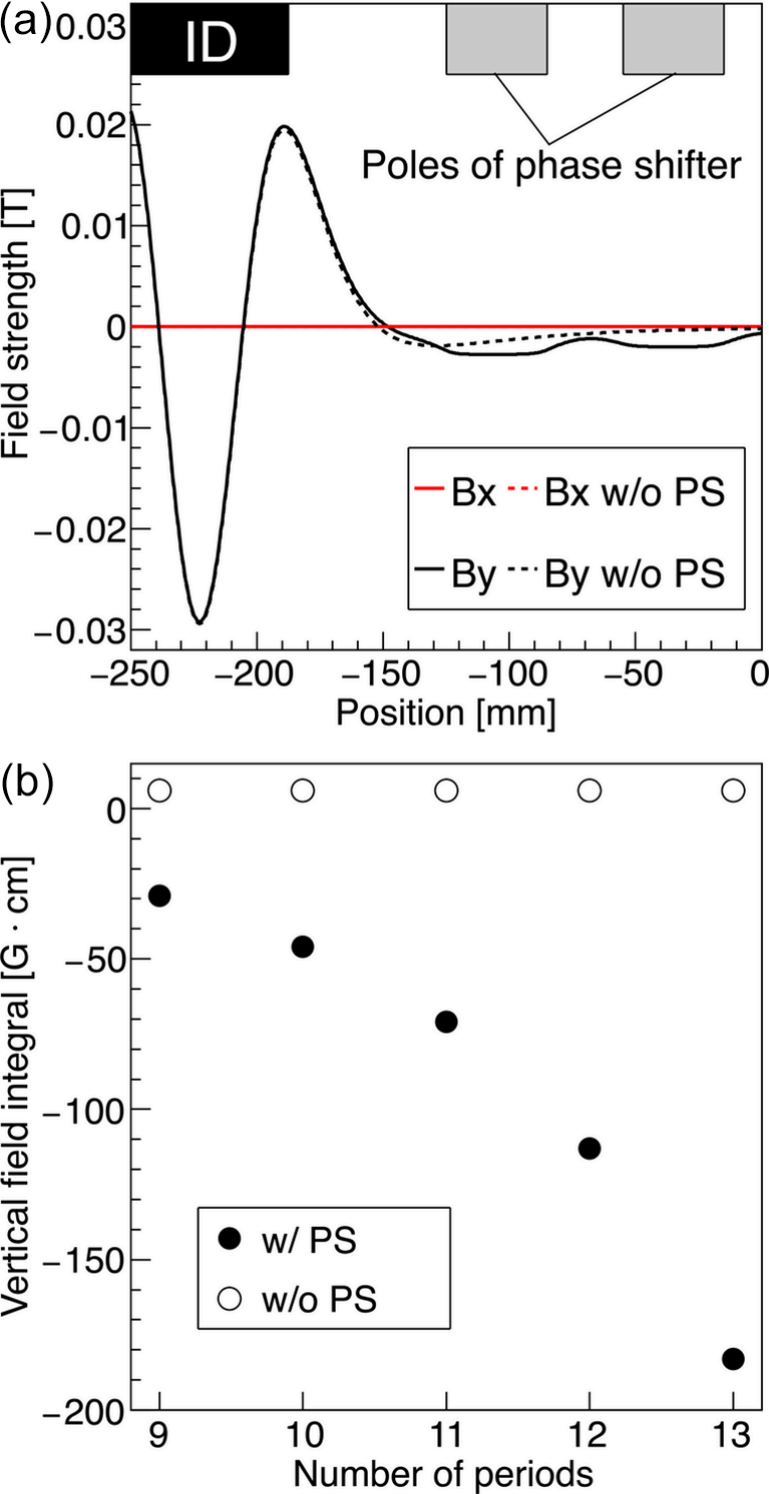
(*a*) Fringe field of the undulator obtained from simulations. The horizontal axis shows the distance along the beam axis. The schematic at the top of the figure depicts the relative position of the undulator magnet array and the poles of the phase shifter. (*b*) Dependence of the vertical field integral on the number of periods obtained by simulations. The undulator gap is 220 mm, and the horizontal linear mode is assumed in the simulation.

Since the space between the undulator segment and the phase shifter is limited, it was necessary to determine the number of periods by taking into account the magnetic interference between the magnet arrays and the phase shifter. Owing to spatial constraints, the maximum number of periods per segment is 13. Fig. 3 ▸(*a*) shows the simulated fringe fields of the undulator segment with 13 periods, for the case of the horizontal linear and the gap of 80 mm as an example. The solid and dashed lines are the results assuming the presence and absence of the phase shifter, respectively. In the case of 13 periods, the distance along the beam axis between the magnets and the poles of the phase shifter becomes as small as 62.5 mm, which leads to magnetic interference where the fringe fields are absorbed by the phase shifter, resulting in an extra field integral. This effect is more pronounced for larger undulator gaps.

In order to mitigate the interference, we evaluated field integrals for different numbers of periods by simulations as shown in Fig. 3 ▸(*b*). The open circles indicate the results for a single undulator segment, while the solid circles show the case where a phase shifter is located upstream of the segment. In the case of a single segment, the field integrals remain constant regardless of the number of periods. On the other hand, when the phase shifter is installed, the field integrals can be reduced by decreasing the number of periods since the distance between the magnets and the phase shifter increases. Although a larger number of periods is desirable from the user’s point of view, we adopted 10 periods to keep the effect of magnetic interference at a level comparable to our adjustment target for the dipole field error of 100 G cm during undulator construction, while also considering the workability to install the phase shifters.

The multipole magnetic fields of four segments in total were measured by the stretched-wire method, and corrections were implemented using magic fingers mounted at the upstream end of the first segment and the downstream end of the last segment. Since the combinations of each segment, such as gap and phase, are extensive, it was not feasible to measure and correct the multipole fields for every configuration. Therefore, the adjustments were carried out for several representative configurations.

**Figure 4:**
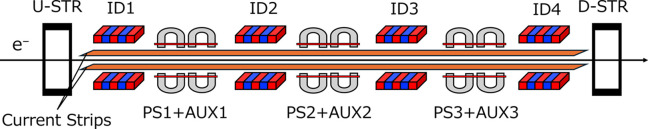
Schematic of devices for orbit correction.

A schematic of the devices used for orbit collection during undulator operation is shown in Fig. 4 ▸. A feed-forward correction remains the orbit discrepancy from the golden orbit within approximately ±10 µm (Obara *et al.*, 2025 ▸). The correction is performed using a combination of steering magnets (U-STR and D-STR), current strips and the auxiliary coils (AUX1, AUX2 and AUX3). Horizontal and vertical steering magnets are installed upstream (U-STR) and downstream (D-STR) of the undulator. The horizontal dipole kick of each undulator segment (ID1, ID2, ID3 and ID4) is corrected using devices located on either side of the segment. That is, U-STR and AUX1 are used for ID1, AUX1 and AUX2 for ID2, AUX2 and AUX3 for ID3, and AUX3 and D-STR for ID4. The vertical kick is corrected using U-STR and D-STR for all the segments. The required current for the corrector magnets is estimated independently for each undulator segment based on the electron beam response. In addition, AUX1, AUX2 and AUX3 locally correct the horizontal kick induced by the excitation of the phase shifter (PS1, PS2 and PS3), respectively. A linear combination of the current for the undulator and the phase shifters derives the final correction current for each corrector magnet. Moreover, current strip wires are attached to the top and bottom of the vacuum chamber to suppress harmful effects on the electron beam due to multipole fields from the undulator. A beam-correction scheme using the current strips and corrector magnets during undulator operation at NanoTerasu can be found in Hosaka *et al.* (2024 ▸).

## 3. Evaluation of performance

**Figure 5:**
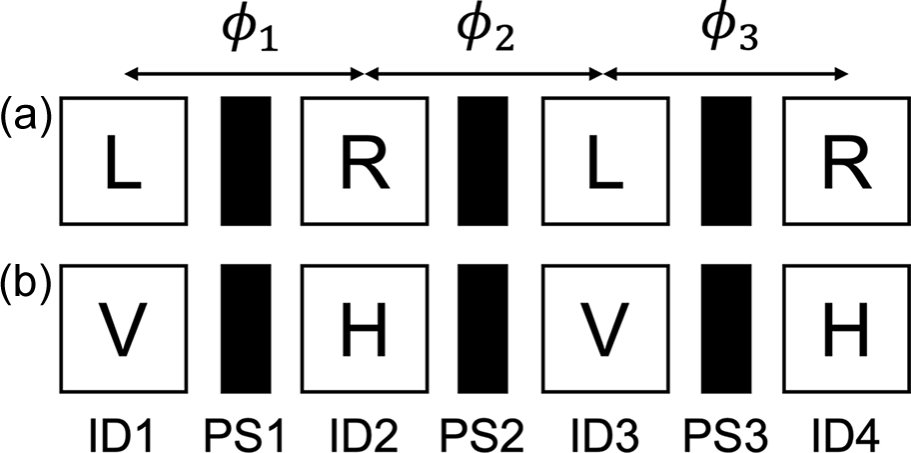
Schematic of a typical configuration of the segmented undulator. Three phase shifters (PS1, PS2 and PS3) are installed in between four APPLE-II segments (ID1, ID2, ID3 and ID4). Each phase shifter controls relative phase (ϕ_1_, ϕ_2_ and ϕ_3_) of adjacent segments. (*a*) LCP and RCP modes are alternately aligned (LR-mode). (*b*) VLP and HLP modes are alternately aligned (VH-mode).

Various polarization configurations are allowed in our undulator by combining the four APPLE-II segments. In the following, we focus on the tandem configurations as employed in previous works (Tanaka & Kitamura, 2002 ▸; Matsuda *et al.*, 2019 ▸). Fig. 5 ▸ shows the two undulator configurations adopted in the present work: (*a*) LCP and RCP modes are arranged in tandem (LR-mode), and (*b*) VLP and HLP modes are arranged in tandem (VH-mode). The relative phases of the SR from the adjacent undulator segments (ϕ_1_, ϕ_2_ and ϕ_3_) are adjusted by the phase shifters installed in between (PS1, PS2 and PS3). In LR-mode, the measured flux *F* is contributed by the LCP radiation from ID1 and ID3 and the RCP radiation from ID2 and ID4 as 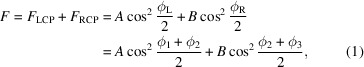
where *A* and *B* are normalizing coefficients. *F*_LCP_ and *F*_RCP_ are the fluxes of the LCP and RCP radiations, which are maximized by satisfying the conditions ϕ_1_ + ϕ_2_ = ϕ_L_ = 2*n*π and ϕ_2_ + ϕ_3_ = ϕ_*R*_ = 2*m*π (*n* and *m* are integers), where ϕ_L_ and ϕ_R_ are relative phases of LCP and RCP radiations, respectively. The flux in VH-mode can also be maximized in the same way.

In this paper, ϕ_2_ is used to control the polarization state of the SR. The polarization states of the SR for LR-mode and VH-mode can be expressed using the normalized Stokes vector as (Tanaka & Kitamura, 2004 ▸) 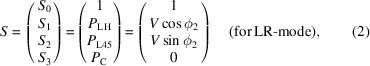

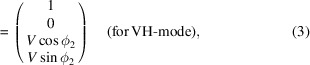
where *P*_LH_, *P*_L45_ and *P*_C_ are the degrees of horizontal linear, 45° linear and circular polarization, respectively. *V* is the degree of polarization. The relation *V* = 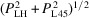 holds for LR-mode, and 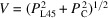 holds for VH-mode. The phase ϕ_2_ causes a transition between two components of the Stokes vector of the SR while keeping *V*. In LR-mode, the transition between *S*_1_ and *S*_2_, namely *P*_LH_ and *P*_L45_, results in a rotation of the polarization azimuth while maintaining the linear polarization. In VH-mode, *S*_2_ and *S*_3_ (*P*_L45_ and *P*_C_) change, leading to a transition between 45/135° linear polarization and left/right circular polarization. During the variation of ϕ_2_, ϕ_1_ and ϕ_3_ are set to keep the conditions for maximizing the flux.

### 3.1. Phase adjustment

**Figure 6:**
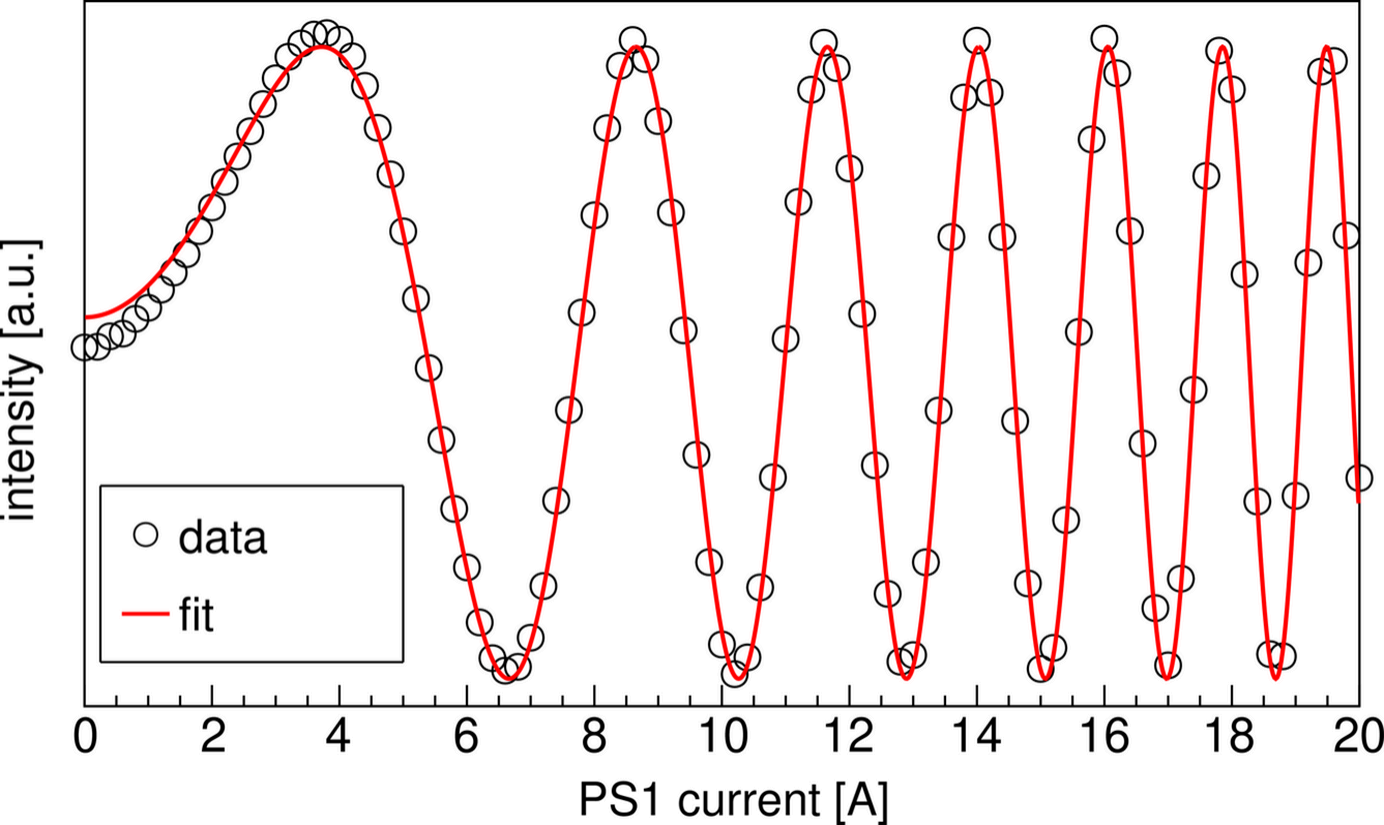
Measured photon flux for *h*ν_1_ = 400 eV in LR-mode while changing the PS1 current. The open circles show the measured flux. The red line shows a fit result. ϕ_L_ is determined to be ϕ_L_ = 0.103*I*^2^ − 1.43 for 400 eV photons.

Here, we describe a typical example of phase adjustment using the phase shifters. Fig. 6 ▸ shows a measured photon flux versus the PS1 current for the fundamental radiation *h*ν_1_ = 400 eV in LR-mode [Fig. 5 ▸(*a*)]. Undulator gaps were adjusted to make the fundamental wavelengths from all segments identical, and the beamline monochromator optics were also tuned to pass the same wavelength. The photon flux was measured by a flux monitor as the drain current of a floated gold plate, located after the exit slit of the monochromator optics (Ohtsubo *et al.*, 2025 ▸). The flux oscillates as the PS1 current increases. This is because *F*_LCP_ changes as ϕ_1_. The phase shift Δϕ_1_ by the chicane field of PS1 *B*(*s*) is obtained as 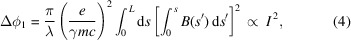
where λ is the wavelength of the SR, *e* the electron charge, γ the Lorentz factor, *m* the electron mass, *c* the speed of light and *L* the length of PS1. Since the magnitude of the chicane field is proportional to the coil current *I*, Δϕ_1_ is proportional to the square of *I*. To obtain the PS1 current for maximizing the flux, fitting was performed using a function 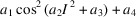, where *a*_1_, *a*_2_, *a*_3_ and *a*_4_ are fitting parameters to be determined to reproduce the measured data in Fig. 6 ▸. The fitting result is shown by a red line in Fig. 6 ▸. The flux oscillation is well reproduced by the fit. We can maximize the flux of LCP radiation using the fitting function, where ϕ_L_ = 2*n*π is considered to be realized. The phase shift is obtained using the PS1 current as Δϕ_1_ = 2*a*_2_*I*^2^, and the relative phase of LCP light as ϕ_L_ = 2*a*_2_*I*^2^ + 2*a*_3_.

Similarly, the PS3 current to hold ϕ_R_ = 2*m*π, and the current dependence of Δϕ_3_ is also evaluated by measuring the flux while changing the PS3 current. On the other hand, PS2 affects the flux of both LCP and RCP radiations as shown in equation (1). However, since the measured flux is well reproduced by the same fitting function, Δϕ_2_ is also obtained as a function of the PS2 current. The same procedure can be used to determine the current to maximize the flux for any wavelengths, including VH-mode, and the correspondence between the phase advance and the excitation current is obtained for each PS.

### 3.2. Energy spectra

**Figure 7:**
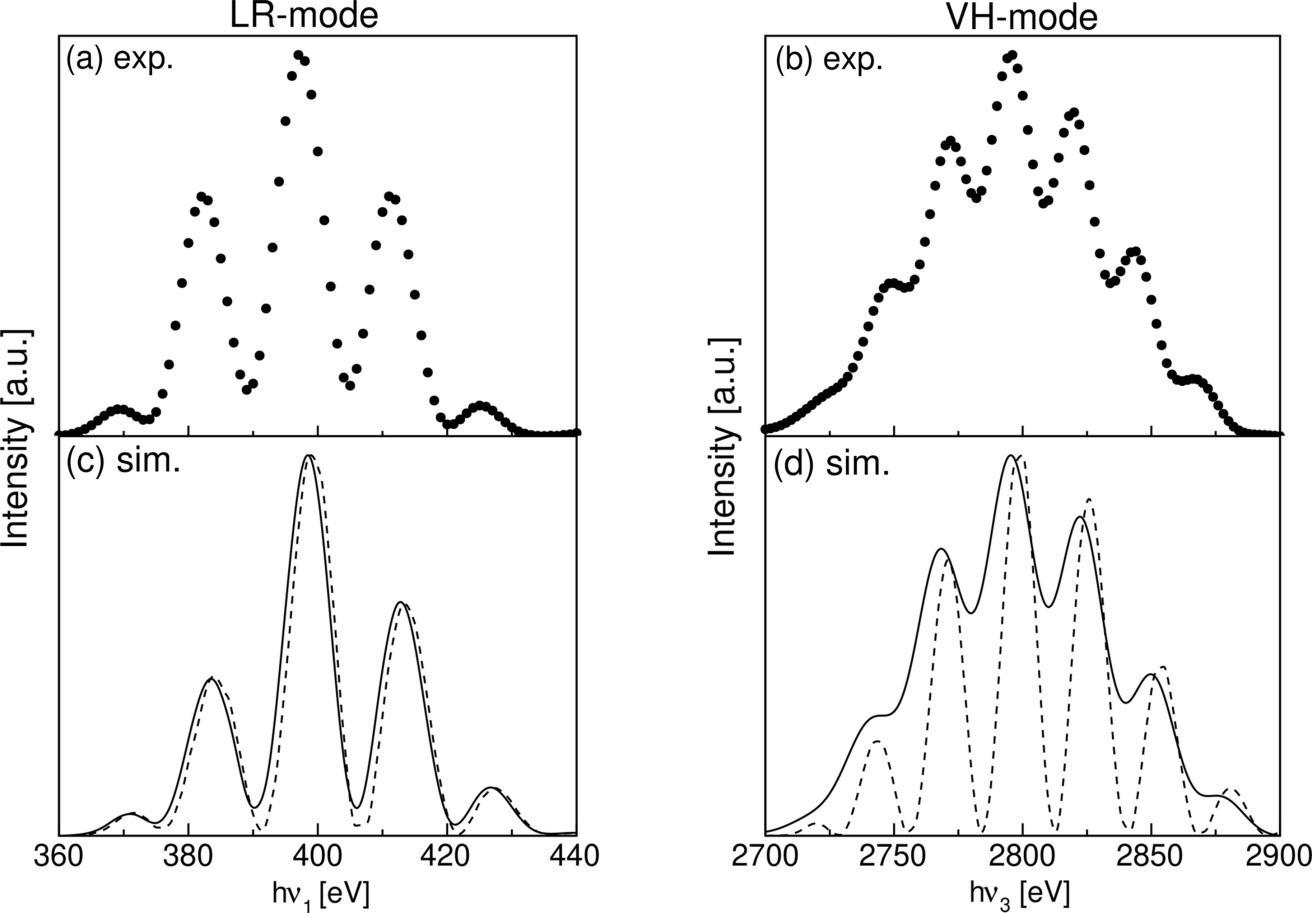
Measured undulator spectra are shown by the dots (*a*) for *h*ν_1_ = 398.6 eV in LR-mode and (*b*) for *h*ν_3_ = 2795 eV in VH-mode. Corresponding simulated spectra are shown by the solid lines in (*c*) and (*d*), whereas the dashed lines show the spectra assuming the zero electron beam emittance and the zero energy spread. The opening size of the FES is 0.1 mm × 0.1 mm for the measured data. The same size is assumed in the simulation.

Fig. 7 ▸(*a*) shows an undulator spectrum for *h*ν_1_ = 398.6 eV measured in LR-mode after maximizing the flux. The flux was measured at the flux monitor, and the photon energy was swept by the monochromator. The front-end slit (FES) is located 14.8 m downstream from the center of the undulator. The opening size of the FES was 0.1 mm (width) × 0.1 mm (height). Three prominent peaks are observed around *h*ν_1_ = 398.6 eV. This multiple-peak structure originates from the constructive interference between LCP lights and between RCP lights, which is a unique feature of segmented undulators (Tanaka & Kitamura, 2002 ▸; Miyawaki *et al.*, 2021 ▸; Matsuda *et al.*, 2019 ▸; Yamamoto *et al.*, 2014 ▸). In addition, we successfully observed the interference of the third harmonic radiation *h*ν_3_ = 2795 eV in VH-mode as shown in Fig. 7 ▸(*b*). One major advantage of introducing the segmented APPLE-II undulator is the availability of higher harmonic interference, thereby expanding the accessible energy range. Circularly polarized third harmonic radiation is expected to be obtained in Fig. 7 ▸(*b*).

Simulated energy spectra corresponding to each measurement using the numerical codes *RADIA* (Chubar *et al.*, 1998 ▸) and *SPECTRA* are shown by the solid lines in Figs. 7 ▸(*c*) and 7 ▸(*d*). Parameters of the storage ring used in the simulation are listed in Table 2 ▸ (Nishimori *et al.*, 2021 ▸; Obara *et al.*, 2025 ▸). Although the peak energies and the intensity ratios of the peaks are slightly different, the number of observed peaks is consistent with the measured spectra. Compared with Fig. 7 ▸(*a*), the intensity ratio between the maxima and minima of the spectral interference pattern is small in Fig. 7 ▸(*b*). The spectra calculated under the assumption of zero emittance and zero energy spread of the electron beam are shown by the dashed lines in Figs. 7 ▸(*c*) and 7 ▸(*d*). It is found that the finite emittance and energy spread broaden the peak widths and cause the peaks to overlap, which leads to a reduction in the intensity contrast between the peaks. Since the higher harmonics are more sensitive to the electron beam emittance and the energy spread, the reduction in contrast is considered to be more pronounced in Fig. 7 ▸(*b*) than in Fig. 7 ▸(*a*).

### 3.3. Polarization measurements

**Figure 8:**
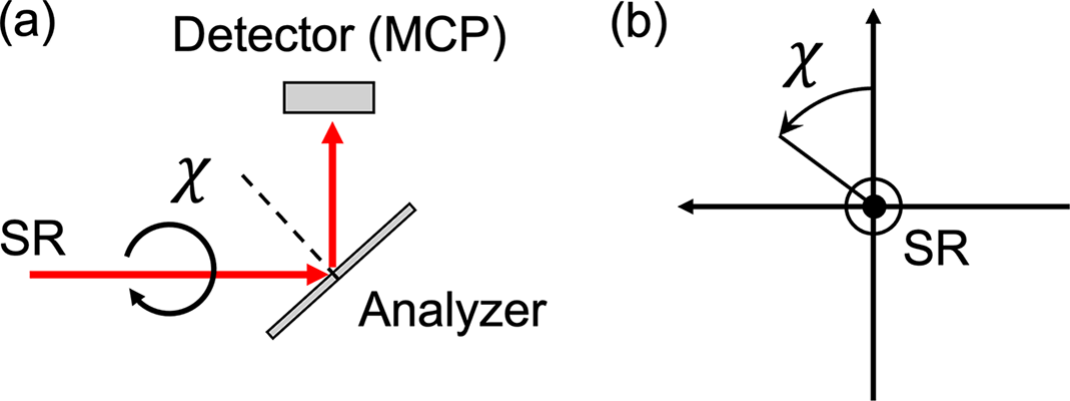
(*a*) Schematic of the polarization measurement. (*b*) Definition of the rotation angle χ.

We measured the degree of linear polarization of SR using ELLI (Kimura *et al.*, 2004 ▸), an apparatus for polarimetry using a rotating analyzer, placed downstream of the monochromator exit slit. Fig. 8 ▸(*a*) shows a schematic of the polarization measurement. SR is reflected by an analyzer and then detected by a microchannel plate (MCP). When the angle of incidence of the SR is equal to pseudo-Brewster’s angle, the analyzer reflects only the *s*-polarized radiation, whose electric field vector is normal to the photon incident plane. The polarization azimuth and the degree of linear polarization of the SR can be determined by measuring the intensity of the reflected light while rotating the azimuthal angle χ of the analyzer against the SR. Fig. 8 ▸(*b*) illustrates the definition of χ used in this paper. The angle χ is defined as the rotation angle measured counterclockwise from the vertical upward direction when an observer faces the beam. As shown in equations (2) and (3), the phase shift Δϕ_2_ changes the polarization azimuth of the SR in LR-mode and the ellipticity angle of the polarization ellipse in VH-mode. The intensity of the reflected light in each mode can be calculated using the Stokes vectors and the Mueller matrices as 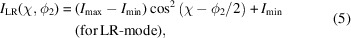

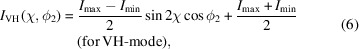
where *I*_max_ and *I*_min_ are the observed maximum and minimum intensities, respectively. Note that, for VH-mode, *I*_max_ is obtained when χ = π/4 + *n*π and ϕ_2_ = 2*m*π, and *I*_min_ is obtained when χ = 3π/4 + *n*π and ϕ_2_ = 2*m*π, where *n* and *m* are integers. The degree of linear polarization *P*_L_ is given by 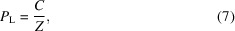
where *C* = (*I*_max_ − *I*_min_)/(*I*_max_ + *I*_min_) is the contrast factor and *Z* = (*R*_*s*_ − *R*_*p*_)/(*R*_*s*_ + *R*_*p*_) is the polarizance of the analyzer. *R*_*s*_ is the reflectivity of the *s*-polarized radiation, whereas *R*_*p*_ is that of the *p*-polarized radiation, whose electric field vector is parallel to the incident plane. In LR-mode, the measured *P*_L_ corresponds to the degree of polarization *V*. In VH-mode, Δϕ_2_ makes a transition between *P*_L45_ and *P*_C_ as shown in equation (3), and *V* corresponds to the measured *P*_L45_ where the intensity of 45° linearly polarized radiation is maximized by varying ϕ_2_.

Polarization measurements were performed at the photon energies 398.6 eV, 835.0 eV and 2795 eV. The analyzers used in the measurements are listed in Table 3 ▸. The analyzers were used at incident angles of approximately 45°, resulting in the reflection of only *s*-polarized radiation. Sc/Cr and Cr/C multilayer analyzers were used for 398.6 eV and 835.0 eV, respectively. The Bragg reflection of Si(111) crystal was used for 2795 eV. Since the reflection angle for Si(111) becomes almost 45°, it can also be used as the polarization analyzer around 2800 eV. The polarizance of the analyzers was evaluated by equation (7) using the single horizontal undulator segment, whose *P*_L_ can be regarded to be unity.

**Figure 9:**
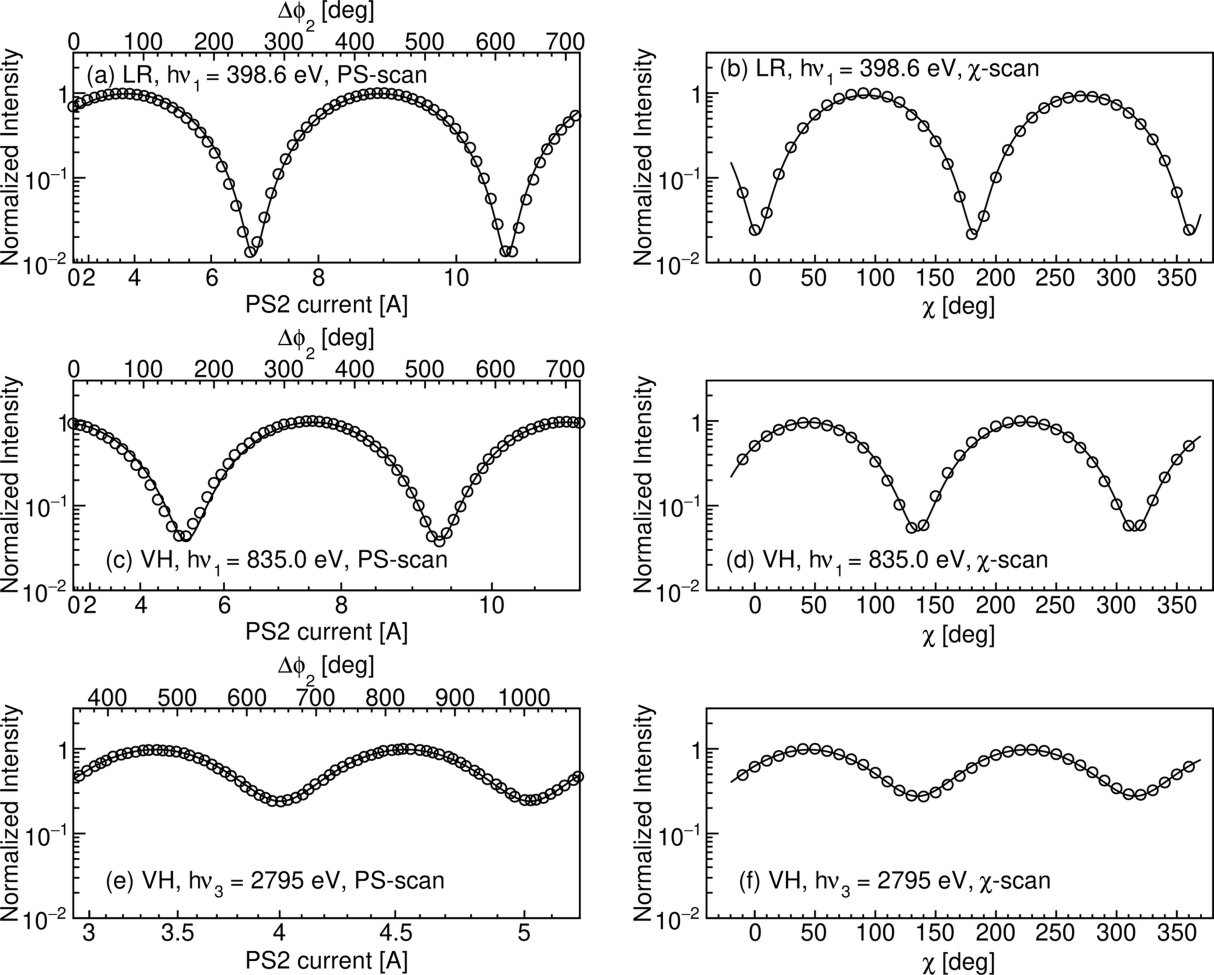
(*a*), (*c*) and (*e*) Normalized photon intensities obtained by varying the phase shift Δϕ_2_. The bottom and top horizontal axes show the PS2 current and corresponding Δϕ_2_, respectively. Δϕ_2_ was obtained using the fitting result as described in Section 3.1. (*b*), (*d*) and (*f*) The fluxes measured by rotating the azimuthal angle χ of the analyzer. The opening size of the FES is 0.1 mm × 0.1 mm. The open circles show the measured data, while the solid lines show the fit results.

Fig. 9 ▸(*a*) shows the measured photon flux by the MCP at *h*ν_1_ = 398.6 eV in LR-mode while varying the PS2 current. The flux was measured with χ fixed at 90°. Since the analyzer is set to be sensitive to VLP photons, VLP (HLP) radiation is considered to be obtained at the phase where the flux reaches its maximum (minimum), and the polarization azimuth changes continuously by sweeping Δϕ_2_. The observed data in Fig. 9 ▸(*a*) agree well with this prediction. By performing a logarithmic curve fitting using equation (5) to the measured data, the contrast factor was determined to be 0.98. On the other hand, the measured photon flux obtained by rotating χ is shown in Fig. 9 ▸(*b*). In this measurement, Δϕ_2_ was adjusted by PS2 so that the polarization azimuth was approximately 90°. The contrast factor for Fig. 9 ▸(*b*) was determined to be 0.96. The degree of polarization and its standard error were evaluated from these two measurements to be *V* = 0.97 ± 0.01, consistent with the simulated value of 0.97. Sufficiently linearly polarized light was obtained through the interference of LCP and RCP light.

Figs. 9 ▸(*c*) and 9 ▸(*e*) show the measured photon flux in VH-mode obtained by rotating ϕ_2_ for the fundamental radiation at *h*ν_1_ = 835.0 eV and the third harmonic at *h*ν_3_ = 2795 eV, respectively. The flux was measured at χ = 45°, sensitive to the inclined linear polarization at 45°. At the phases where the flux reaches its maximum and minimum, 45° and 135° inclined linear polarized light are generated. Since the ellipticity angle is varied by the phase shift, the circularly polarized light is considered to be obtained at the phase halfway between the flux maximum and minimum. The measured photon fluxes by rotating χ for 835.0 eV and 2795 eV are shown in Figs. 9 ▸(*d*) and 9 ▸(*f*), respectively. ϕ_2_ was adjusted to generate linearly polarized light at 45° for the measurements. The degrees of polarization estimated from the measured contrast factors using equation (6) were evaluated to be *V* = 0.91 ± 0.01 for 835.0 eV and *V* = 0.59 ± 0.03 for 2795 eV. On the other hand, the simulated *V* are 0.93 (835.0 eV) and 0.70 (2795 eV). Although the measured *V* for the third harmonic is lower than that for the fundamental, the ellipticity angles were successfully controlled by the phase shifter for both the fundamental and the third harmonic by combining VLP and HLP radiations. The reduced polarization in the third harmonic is discussed in the next section.

Flux measurements with rotating ϕ_2_ were also performed for the side peaks located at 2820 eV and 2844 eV [see Fig. 7 ▸(*b*)], and the polarization degrees were found to be 0.67 and 0.70, respectively. The simulation predicts the corresponding values of 0.76 and 0.78. These results show that a higher degree of polarization can be achieved in the side peaks than in the main peak, which is consistent with the previous numerical studies and experiments (Yamamoto *et al.*, 2014 ▸; Matsuda *et al.*, 2019 ▸; Miyawaki *et al.*, 2021 ▸). Results of the polarization measurements are summarized in Table 4 ▸.

### 3.4. Spatial dependence

**Figure 10:**
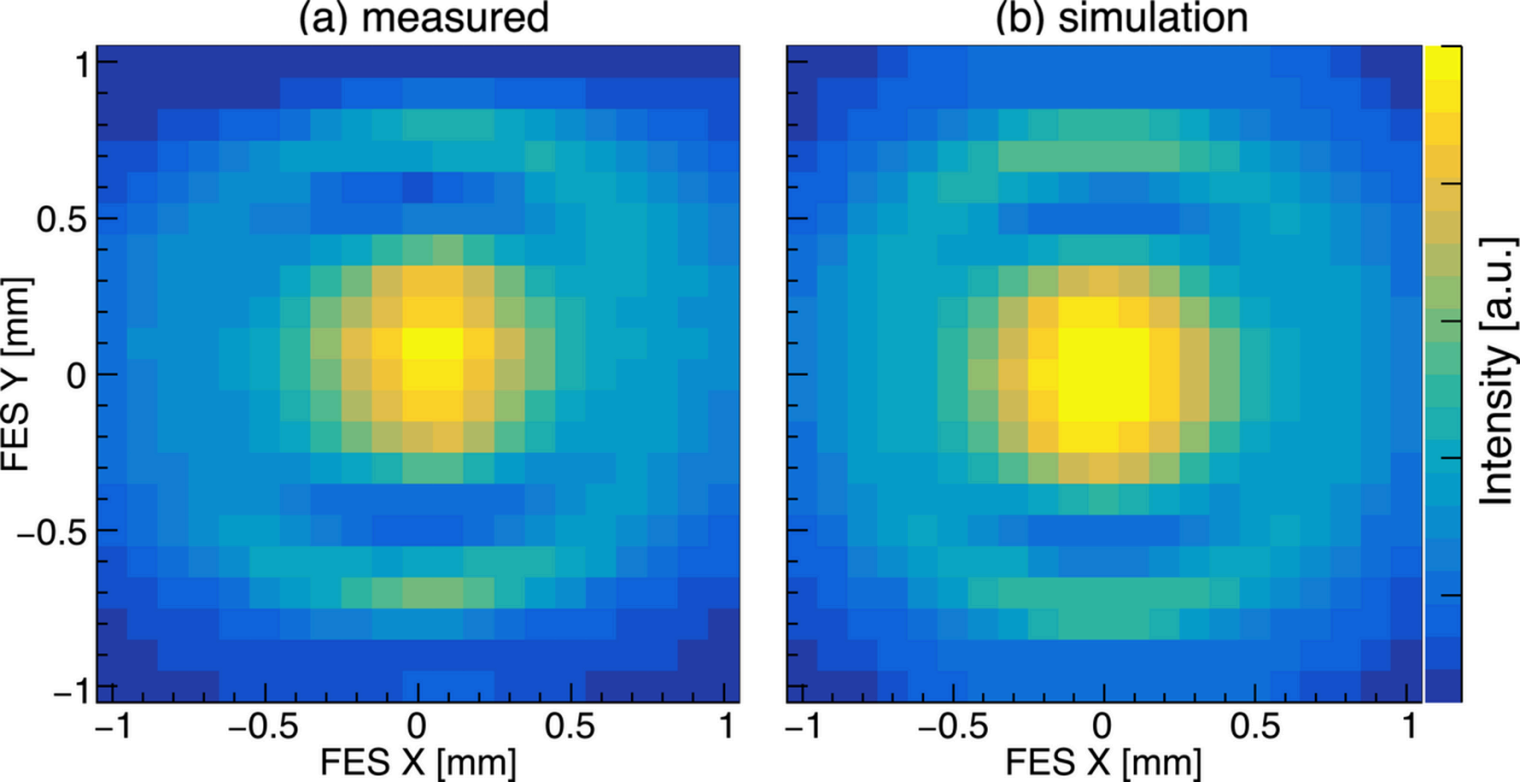
(*a*) Position dependence of photon flux measured for *h*ν_1_ = 398.6 eV in LR-mode. (*b*) Simulated result shown for comparison. The opening size of the FES is 0.1 mm × 0.1 mm. The measurement was performed by sweeping the center position of the FES in 0.1 mm steps.

The flux and degree of polarization of SR from the segmented undulator exhibit characteristic spatial dependence. We performed a 2D scan of the FES and measured the photon flux using the mirror current downstream of the monochromator, which is proportional to the monochromated photon flux. Fig. 10 ▸(*a*) shows a spatial dependence of the flux measured in LR-mode at *h*ν_1_ = 398.6 eV. The distribution has a concentric pattern with the central region being the brightest, and light and dark fringes are observed in the vertical direction. This feature is consistent with the simulated result shown in Fig. 10 ▸(*b*). The phase shifters are tuned to maximize the flux on the axis. As the observation position changes, variations in the optical path lengths from each undulator segment lead to corresponding phase differences, resulting in the interference pattern. The contrast in the horizontal direction is considered to be less distinct due to the large horizontal spread of the electron beam (Obara *et al.*, 2025 ▸).

**Figure 11:**
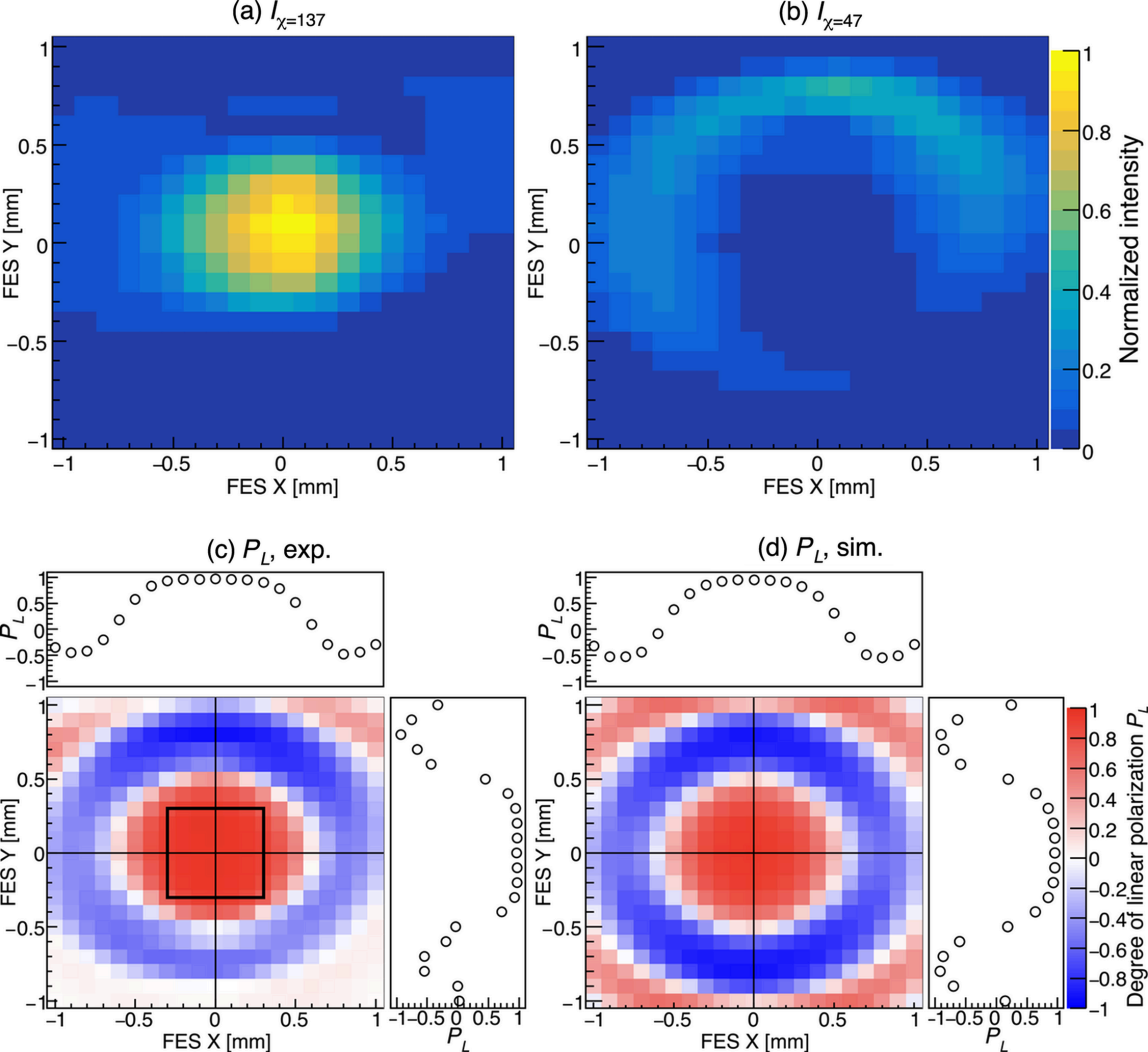
Position dependence of measured flux at (*a*) χ = 137° and (*b*) χ = 47° for *h*ν_1_ = 398.6 eV in LR-mode. The Sc/Cr multilayer was used for the analyzer. The opening and sweep step of the FES are the same as Fig. 10 ▸. (*c*) Degree of linear polarization *P*_L_ obtained from (*a*) and (*b*). The top and right graphs show the position dependence of *P*_L_ along *Y* = 0 and *X* = 0 axes drawn by the solid lines in the contour map, respectively. The open square shows the FES aperture of 0.6 mm × 0.6 mm. (*d*) Simulated *P*_L_.

According to the polarization measurement using ELLI, the polarization azimuth of the observed linearly polarized light at the position (*X*, *Y*) = (0, 0) mm was found to be 137°. We note that the polarization azimuth can be arbitrarily controlled via PS2 as discussed in the previous section. We measured the photon flux of the SR reflected by the analyzer while performing FES scans. Fig. 11 ▸(*a*) shows a spatial distribution of the flux *I*_χ=137_ measured by the analyzer azimuth χ fixed at 137°, whereas Fig. 11 ▸(*b*) shows the flux *I*_χ=47_ measured at χ = 47°. The Sc/Cr analyzer in Table 3 ▸ was used for the measurements. In contrast to *I*_χ=137_, *I*_χ=47_ has enhanced intensity in the outer region rather than the center. A map of the contrast factor *C* = (*I*_χ=137_ − *I*_χ=47_)/(*I*_χ=137_ + *I*_χ=47_) was obtained from these two distributions. The degree of linear polarization *P*_L_ was evaluated by *P*_L_ = *C*/*Z*, and its spatial distribution is shown in Fig. 11 ▸(*c*). The red regions in the figure correspond to large *I*_χ=137_ and the blue regions to large *I*_χ=47_. A concentric interference pattern is observed. *P*_L_ at the position (*X*, *Y*) = (0, 0) mm is 0.96, indicating that highly polarized photons are obtained on axis. As the observation position moves outward, the degree of polarization decreases, and the polarization is reversed in the outer ring region. By comparing Figs. 10 ▸(*a*) and 11 ▸(*c*), it is found that the polarization states of the light at the center and in the outer ring in Fig. 10 ▸(*a*) are orthogonal to each other. The formation of the concentric interference pattern is one of the unique characteristics exhibited by the segmented undulator. Fig. 11 ▸(*d*) shows the distribution of simulated *P*_L_. The simulation also reproduces the concentric variation in polarization.

**Figure 12:**
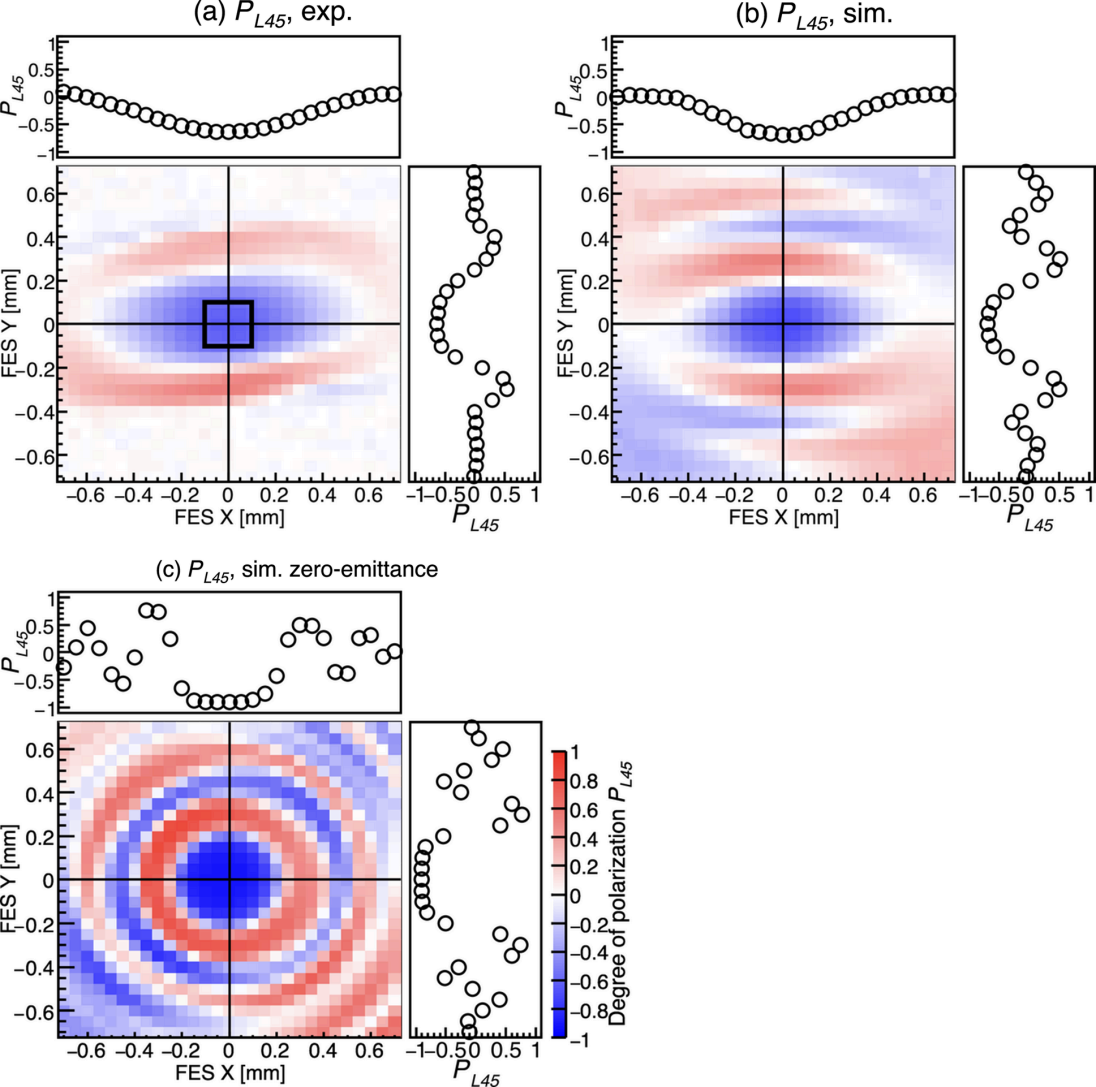
Position dependence of the degree of linear polarization at 45° *P*_L45_ for *h*ν_3_ = 2975 eV in VH-mode. Aperture size of the FES is 0.1 mm × 0.1 mm. Sweep step of the scan is 0.05 mm. The top and right graphs of the contour maps show the position dependence of *P*_L45_ along *Y* = 0 and *X* = 0 axes drawn by the solid lines. (*a*) Experimental *P*_L45_. The open square corresponds to the FES aperture of 0.2 mm × 0.2 mm. (*b*) Simulated *P*_L45_. (*c*) Simulated *P*_L45_ under the assumption of the zero electron beam emittance.

We also performed similar measurements for the third harmonic radiation at *h*ν_3_ = 2795 eV in VH-mode. The Si(111) crystal in Table 3 ▸ was used for the analyzer. PS2 was adjusted so that the reflected intensity from the analyzer was maximized at χ = 135°. Spatial distributions of *I*_χ=45_ and *I*_χ=135_ were measured by the FES scan. Fig. 12 ▸(*a*) shows a map of the measured degree of linear polarization at 45° *P*_L45_, evaluated by *P*_L45_ = *C*/*Z* = (*I*_χ=45_ − *I*_χ=135_)/(*I*_χ=45_ + *I*_χ=135_)/*Z*. A concentric interference pattern with spatially varying polarization is observed; however, it appears horizontally expanded compared with Fig. 11 ▸(*c*). Fig. 12 ▸(*b*) shows the simulated *P*_L45_, where the horizontally expanded pattern is also reproduced. On the other hand, Fig. 12 ▸(*c*) shows the spatial distribution of *P*_L45_ simulated under the assumption of the zero electron beam emittance. Although the vertical size of the central blue region remains nearly constant in Figs. 12 ▸(*a*)–12 ▸(*c*), the horizontal size becomes smaller in Fig. 12 ▸(*c*). These calculations show that the horizontal expansion of the interference pattern can be attributed to the electron beam emittance. In addition, a comparison between Figs. 11 ▸(*c*) and 12 ▸(*a*) shows that the vertical size of the central circular or elliptical region is smaller in Fig. 12 ▸(*a*). This is because a shorter-wavelength SR exhibits a larger phase difference for a given optical path difference. The on-axis degrees of polarization in Figs. 12 ▸(*a*)–12 ▸(*c*) are *P*_L45_ = −0.64, −0.70 and −0.90, respectively, indicating that the achievable *P*_L45_ is also degraded by the finite emittance. As also shown in Table 4 ▸, the experimentally obtained polarization degrees for the third harmonic are lower than the simulated results. The simulations do not include phase errors of the undulator and misalignments of the optical axes of each undulator segment. A fine adjustment of the horizontal axes of each undulator segment using the auxiliary coils might improve the achievable polarization degrees.

**Figure 13:**
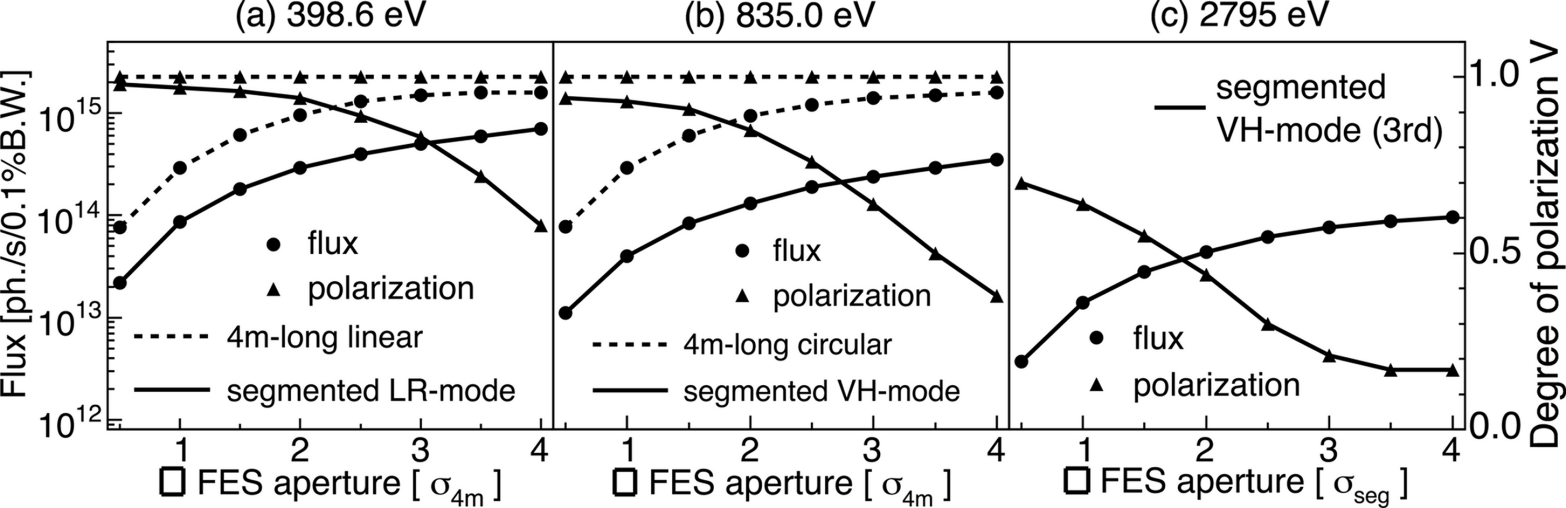
Dependence of the flux and the degree of polarization on the FES aperture obtained from simulations for (*a*) *h*ν_1_ = 398.6 eV, (*b*) *h*ν_1_ = 835.0 eV and (*c*) *h*ν_3_ = 2795 eV. The circles indicate the flux, and the triangles indicate the degree of polarization. The solid lines represent the segmented undulator, while the dashed lines show the results for a single 4 m-long APPLE-II undulator (the periodic length: 56 mm, the number of periods: 71). The horizontal axes show the FES aperture normalized by the photon beam size in units of □σ = σ_*x*_ × σ_*y*_, where σ_*x*_ and σ_*y*_ are the horizontal and vertical beam size at the FES. □σ_4m_ is for the 4 m-long undulator defined as (*a*) 0.35 mm × 0.30 mm and (*b*) 0.27 mm × 0.21 mm, whereas □σ_seg_ for the segmented undulator as (*c*) 0.32 mm × 0.21 mm.

Finally, we discuss the photon flux expected from the segmented undulator. The solid lines in Figs. 13 ▸(*a*)–13(*c*) show the dependence of the simulated flux and degree of polarization on the FES aperture for *h*ν_1_ = 398.6 eV, 835.0 eV and *h*ν_3_ = 2795 eV, respectively. While increasing the aperture allows for a higher photon flux, it also leads to a reduction in the polarization degree. This behavior originates from the spatial dependence of the polarization. For comparison, the relationships between the flux and the degree of polarization of a single 4 m-long APPLE-II undulator (the periodic length: 56 mm and the number of periods: 71) are shown by the dashed lines. In the case of the single undulator, it is optimal to set the aperture to approximately 4σ_*x*_ × 4σ_*y*_ of the photon distribution at the FES (denoted □4σ_4m_ in Fig. 13 ▸). On the other hand, for the segmented undulator, it is necessary to determine a reasonable aperture that balances both the flux and the polarization degree by performing measurements or simulations corresponding to Figs. 11 ▸–13 ▸ ▸. For example, the open square in Fig. 11 ▸(*c*) shows the aperture size of 0.6 mm × 0.6 mm. Setting the aperture to this size enables an XMLD experiment using SR with the polarization degree of 0.95. Similarly, the open square in Fig. 12 ▸(*a*) shows a size of 0.2 mm × 0.2 mm. Assuming that *P*_C_ = *P*_L45_, an XMCD experiment in the tender X-ray region using a polarization degree of 0.59 is available. Compared with a single 4 m-long undulator, the flux of the segmented undulator is about one order of magnitude lower. However, the ability to provide circularly polarized light with wide energy coverage as well as fast polarization switching makes this undulator an attractive option.

## 4. Summary and outlook

We have newly developed the segmented undulator consisting of four APPLE-II undulators and three electromagnetic phase shifters. The undulator was adopted for NanoTerasu BL13U, which was designed for X-ray absorption spectroscopy over a wide energy range of 180–3000 eV. According to the polarization measurements using the rotating analyzers, a high degree of linear polarization *V* = 0.97 ± 0.01 was obtained by the superposition of LCP and RCP radiations at *h*ν_1_ = 398.6 eV. The polarization azimuth was controlled over a 2π angular range using the phase shifters. For the combination of VLP and HLP radiations, the degree of polarization for the fundamental at *h*ν_1_ = 835.0 eV and for the third harmonic at *h*ν_3_ = 2975 eV were *V* = 0.91 ± 0.01 and 0.59 ± 0.03, respectively, which were measured by fixing the azimuthal angle of the analyzer at 45°. The ellipticity angles were successfully controlled by the phase shifters for both the radiations. Although circular polarization was not directly measured, circularly polarized light of the third harmonic is considered to be available in addition to the fundamental, thereby extending the accessible energy of circularly polarized light from the soft to tender X-ray region.

The spatial distributions of the flux and the degree of polarization of the SR were also measured. Concentric patterns due to the interference of the SR emitted from each undulator segment were observed in the distributions. The concentric interference was well reproduced by the simulations. By measuring or simulating the polarization map, an appropriate FES aperture that balances both the flux and the polarization degree can be determined.

The present work suggests that this undulator is useful for the XMCD/XMLD measurements with lock-in techniques. A switching speed of polarization has so far been limited to ∼ 0.1 Hz for the stable operation of the storage ring. This limitation is due to insufficient power supplies and control systems for the phase shifters and auxiliary coils; however, these are currently undergoing improvements. As the first step, a 10 Hz switching will be realized in the near future.

## Figures and Tables

**Table 1:** Principal parameters of the segmented APPLE-II undulator

Periodic length	56 mm
Number of periods	10 × 4
Minimum gap	15 mm
Maximum deflection parameters	3.3 (vertical linear)
	4.7 (horizontal linear)
	3.8 (circular)
Magnet material	TiN-coated NdFeB
Remnant induction	≥ 1.29 T
Coercivity, *H*_*cj*_	≥ 24.2 kOe

**Table 2:** Parameters of the NanoTerasu storage ring used in the simulation

Energy	2.998 GeV
Beam current	400 mA
Natural emittance	1.14 nm rad
Coupling constant	2.1%
Energy spread	0.0972%
Betatron functions (β_*x*_, β_*y*_)	13.0 m, 3.0 m
Dispersion functions (η_*x*_, η_*y*_)	0.0 m, 0.0 m

**Table 3:** List of analyzers

*h*ν (eV)	Material	*d* (nm)	*N* (layers)	Polarizance
398.6	Sc/Cr	2.24	200	0.997
835.0	Cr/C†	3.18	200	0.995
2795	Si(111)	0.314	Crystal	0.999

† The third reflection was used.

**Table 4:** Results of the polarization measurements

*h*ν	Mode	*C* (PS-scan)	*C* (χ-scan)	*V* (exp.)	*V* (sim.)
398.6 eV	LR	0.98	0.96	0.97 ± 0.01	0.97
835.0 eV	VH	0.92	0.90	0.91 ± 0.01	0.93
2795 eV	VH	0.61	0.56	0.59 ± 0.03	0.70
2820 eV	VH	0.67	–	0.67	0.76
2844 eV	VH	0.70	–	0.70	0.78

## Data Availability

All the data shown in this article are available upon request.
